# Towards the Improved Accuracy of Hepatitis E Diagnosis in Vulnerable and Target Groups: A Global Perspective on the Current State of Knowledge and the Implications for Practice

**DOI:** 10.3390/healthcare9020133

**Published:** 2021-01-29

**Authors:** Jasminka Talapko, Tomislav Meštrović, Emina Pustijanac, Ivana Škrlec

**Affiliations:** 1 Faculty of Dental Medicine and Health, Josip Juraj Strossmayer University of Osijek, HR-31000 Osijek, Croatia; jtalapko@fdmz.hr; 2 University Centre Varaždin, University North, HR-42000 Varaždin, Croatia; tmestrovic@unin.hr; 3 Clinical Microbiology and Parasitology Unit, Dr. Zora Profozić Polyclinic, HR-10000 Zagreb, Croatia; 4 Faculty of Natural Sciences, Juraj Dobrila University of Pula, HR-52100 Pula, Croatia; emina.pustijanac@unipu.hr

**Keywords:** hepatitis E, hepatitis E virus, pregnancy, diagnosis

## Abstract

The hepatitis E virus (HEV) is a positive single-stranded, icosahedral, quasi-enveloped RNA virus in the genus *Orthohepevirus* of the family *Hepeviridae*. *Orthohepevirus* A is the most numerous species of the genus *Orthohepevirus* and consists of eight different HEV genotypes that can cause infection in humans. HEV is a pathogen transmitted via the fecal–oral route, most commonly by consuming fecally contaminated water. A particular danger is the HEV-1 genotype, which poses a very high risk of vertical transmission from the mother to the fetus. Several outbreaks caused by this genotype have been reported, resulting in many premature births, abortions, and also neonatal and maternal deaths. Genotype 3 is more prevalent in Europe; however, due to the openness of the market, i.e., trade-in animals which represent a natural reservoir of HEV (such as pigs), there is a possibility of spreading HEV infections outside endemic areas. This problem is indeed global and requires increased hygiene measures in endemic areas, which entails special care for pregnant women in both endemic and non-endemic regions. As already highlighted, pregnant women could have significant health consequences due to the untimely diagnosis of HEV infection; hence, this is a population that should be targeted with a specific combination of testing approaches to ensure optimal specificity and sensitivity. Until we advance from predominantly supportive treatment in pregnancy and appraise the safety and efficacy of a HEV vaccine in this population, such screening approaches represent the mainstay of our public health endeavors.

## 1. Introduction

Hepatitis E virus (HEV) is a positive single-stranded, small, quasi-enveloped RNA virus. It is the only member of the genus *Orthohepevirus* of the family *Hepeviridae* [1,2]. *Orthohepevirus A* is the largest species of the genus *Orthohepevirus* and consists of eight different genotypes of HEV that can cause infection in humans (HEV-1, 2, 3, 4, and 7), rabbits (HEV-3), pigs (HEV-3 and 4), boars (HEV-3, 4, 5, and 6), deer (HEV-3), mongoose (HEV-3), camel (HEV-7 and HEV-8), and yak (HEV-4) [3,4], as shown in Table 1.

In northern India, during the 1978 hepatitis epidemic, the hepatitis E virus was first identified in the Kashmir Valley. In that specific outbreak there were 5200 cases of hepatitis, resulting in 17,000 deaths [5].

The structure of the HEV genome was thoroughly studied in 1991. The RNA molecule consists of 7200 positively directed nucleotides [6]. There are three main coding regions whose protein products have not been thoroughly studied. It was also found that there is a fourth coding region characteristic of the HEV-1 genotype [6,7,8]. The heparan sulfate proteoglycan, HSC70 (heat shock cognate 70 kDa), allows HEV to enter the cell. Other specific, but insufficiently studied, receptors and co-receptors are also crucial for entering the cell. The process of releasing, transcribing, and translating HEV RNA, as well as the assemblage of new virions, occurs in the cytoplasm [6,9,10].

## 2. Molecular Biology of the Hepatitis E Virus

**Figure 1:**
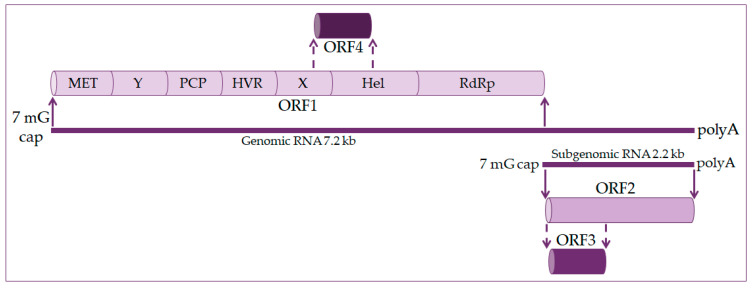
Hepatitis E virus genome organization. The genome is 7.2 kb positive-strand RNA with a 5′ 7-methylguanosine cap (7 mG cap) and a 3′ polyadenylated tail (polyA). It harbors four open reading frames (ORF). ORF1 codes nonstructural proteins, including a methyltransferase (MET), an RNA helicase (Hel), and an RNA-dependent RNA polymerase (RdRp), as well as less well-characterized domains, such as the Y domain, a papain-like cysteine protease (PCP), a hypervariable region (HVR), and the X domain. ORF2 and ORF3 encode the viral capsid and a small protein involved in virus secretion, respectively, translated from a 2.2 kb subgenomic RNA generated during viral replication. The ORF4 is found in the HEV-1 genotype and codes protein enhanced viral replication.

The hepatitis E virus genome is a single-stranded positive linear RNA 7.2 kb in length [11,12,13]. HEV RNA consists of four open reading frames (ORF) and untranslated regions at the 5’ and 3’ ends. At the 5’ end is a 7-methylguanosine cap and at the 3’ end is a polyadenylated tail, just like eukaryotic messenger RNA (mRNA) [14,15], as shown in Figure 1. During viral replication two viral RNA are generated: a full-length RNA of 7.2 kb and subgenomic RNA of 2.2 kb [13,14].

ORF1 occupies about 2/3 of the genome and codes nonstructural proteins included in viral RNA replication. These are methyltransferase (MET), X domain, helicase (Hel), and RNA-dependent RNA polymerase (RdRp). The function of other domains such as the Y domain, papain-like cysteine protease (PCP), and hypervariable region (HVR) is still undetermined [16,17].

ORF2 encodes for the capsid protein and has its role in HEV infection diagnostics and vaccine development. It is a 72 kDa protein that is N-glycosylated in mammalian cells [17,18].

ORF3 codes a small phosphoprotein (VP13) of 13 kDa and is phosphorylated at serine residue [16,18]. The ORF2 and ORF3 proteins are the results of subgenomic RNA translation [19].

ORF4 is unique to the HEV-1 genotype. It is entirely within ORF1 and overlaps with the X domain and helicase. Transcription of ORF4 protein is controlled by an internal ribosome entry site (IRES)-like RNA structure, and it plays a vital role in HEV RNA polymerase’s proper functioning. The ORF4 protein promotes viral RdRp activity and viral replication [14,18,20].

**Figure 2:**
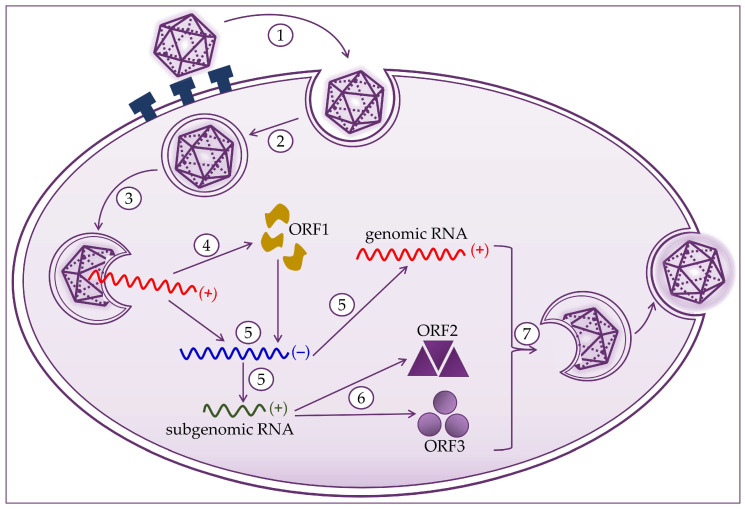
Hepatitis E virus life cycle consists of (1) attachment to heparan sulfate proteoglycans and entry to host cell; (2) clathrin-dependent endocytosis; (3) release of the positive-strand RNA genome into the cytosol; (4) translation to produce the ORF1 proteins; (5) replication via a negative-strand RNA mediator and synthesis of genomic and 2.2 kb subgenomic RNAs; (6) translation of the subgenomic RNA to produce the ORF2 and ORF3 proteins; and (7) packaging, virion assembly, and release of the newly formed virus.

Hepatocytes are target cells where HEV replication takes place, as shown in Figure 2. HEV binds to the cell membrane through interactions with heparan sulfate proteoglycans, and enters the cell via clathrin-dependent endocytosis [21,22,23]. The virus genome is released into the cytoplasm, where viral RNA replication ensues. Positive-strand genomic RNA of 7.2 kb acts as messenger RNA for the translation of nonstructural ORF1 proteins. Based on positive-strand genomic viral RNA, a negative-strand RNA is synthesized, which subsequently serves as a template for synthesizing positive-strand 7.2 kb viral RNA and 2.2 kb subgenomic RNA [20,21,24]. Subgenomic RNA acts as a mRNA for the translation of ORF2 and ORF3 proteins. Translation of the capsid proteins and phosphoproteins occurs on the ribosomes of the host cell [1,19,23,24]. The next step is to assemble the virus and release the newly produced virions. Packaging of the viral genome in the capsid occurs spontaneously by the assembly of the non-glycosylated capsid proteins. The formation of a quasi-enveloped particle involves the phosphorylation of the VP13 protein. Virion particles become intracellular vesicles by the interaction of VP13 proteins with cell sorting host proteins. They are then released from the cell and can readily enter the bloodstream [11,19,20,21,25].

## 3. Epidemiology of HEV

HEV is a major global health problem in developing and developed countries and is mostly zoonotic [11,26,27]. The World Health Organization (WHO) estimates that the incidence of HEV infections is 20 million annually, leading to approximately 3.3 million symptomatic cases of hepatitis E [28]. In 2015, it caused about 44,000 deaths, representing 3.3% of deaths due to viral hepatitis [28,29].

In the last two decades, the view that hepatitis E is a self-limiting tropical disease without a significant presence in developed countries has been revised because HEV has been found to circulate in Western countries [30,31]. It was noticed that HEV’s epidemiology is more complicated than it was initially thought. Namely, there are two different epidemiological forms of HEV [32,33,34]. Outbreaks of HEV-1 and HEV-2 occur periodically in several regions of Asia, Africa, Mexico, and the Middle East due to floods causing water supplies contamination with feces. The first HEV epidemic (1955–1956) infected 29,300 people in India [14,35,36]. In hyperendemic regions of the world, the infection is caused by HEV genotypes 1 and 2, most often in young adults, and extremely severe forms develop in pregnant women [17,37,38].

Genotype 1 causes infections in Asia (south, southeast, and central) and North Africa [17,39]. Furthermore, the presence of the HEV-1 genotype was observed in Cuba and Venezuela [32]. The HEV-1 genotype is a common cause of acute hepatitis E in Europe, introduced by international travelers, mostly from Asia [32]. The HEV-2 genotype was first detected in an epidemic in Mexico but was later seen in African countries (Chad, Central African Republic, Egypt, Democratic Republic of Congo, Nigeria, and Namibia) [40]. HEV-3 and HEV-4 infections are endemic in developed countries. They are mainly transmitted from animals or occur due to consuming contaminated food, and parenteral transmission via blood transfusion has been reported [11,13,41]. Genotype 3 is common among pigs worldwide, and they are a significant reservoir for human infections [13,42,43]. It has also been found in shellfish in Scotland and southern Italy [44]. Strains within genotype 4 are similar to genotype 3 [32], and they are widespread in South and East Asia [13].

The prime reservoir of hepatitis E are domestic pigs and wild boars [44]. Infections are more common in people over the age of 50, and recent studies have shown that infections are more common in men than in women [13,45]. They cause sporadic acute cases of hepatitis E in the United States, Europe, China, and Japan [40]. Genotypes 5 and 6 were recorded only in wild boars, and genotypes 7 and 8 were identified in camels and camelids [6], as shown in Table 2.

It is estimated that HEV infections caused 70,000 deaths due to acute liver failure and about 3000 stillbirths [17]. China is considered an endemic HEV region [46]. Contact with cats and pigs, age, and soil exposure is associated with HEV infection; furthermore, studies have shown a high prevalence of HEV and significant potential for HEV infection transmission in pregnant women in China [47]. In the last ten years, more than 21,000 acute clinical cases of hepatitis E have been reported in European countries, with 28 deaths. The majority (80%) of patients are in Germany, France, and the United Kingdom [48]. The prevalence of HEV antibodies in England was 13%, increased over the years, and reached 25% in individuals over 50 years of age. Based on the incidence assessment, it has been proven that common risk factors still exist for acquiring HEV infection in all age groups in England. Current estimates of HEV incidence revealed that the incidence did not differ in different age groups [49]. Based on research conducted in England, excessive alcohol consumption and diabetes have been observed among people with acute hepatitis E, which are risk factors for liver fibrosis and steatosis [50].

Hepatitis E is a proven autochthonous zoonosis in Croatia caused by *Orthohepevirus* A genotype 3 (HEV-3) [51]. In Croatia, a seroprevalence of 32.94% in domestic pigs was found in 11 of 14 counties. With seropositive wild boars found in six of the 16 counties, the seroprevalence was 31.10%. The highest seroprevalence was found in Vukovar-Srijem and Osijek-Baranja counties (eastern Croatia), where pig breeding dominates and where wild boars’ highest density was recorded [52,53]. It has been proven that all detected HEV strains in Croatia are genetically closely related to strains found in humans and/or animals from other European countries. All of the above indicates that live animals’ trafficking or the wild boars’ movement increases HEV infection risk [54].

## 4. HEV Infections in Pregnant Women

The first symptomatic cases of HEV infections in pregnant women were reported in Nepal in 1987. New infection cases have been reported in developing countries in refugee camps (Kenya, Somalia, Uganda, and Sudan) [55].

There is growing evidence that HEV is an essential factor in maternal morbidity and mortality in South Asia, mostly if infection occurs in the third trimester with genotype 1 [5]. A high rate of IgG anti-HEV seroprevalence was found in pregnant women in Addis Ababa, Ethiopia [56]. In endemic regions and sub-Saharan Africa and South Asia, mortality among pregnant women is often 30% or higher. HEV infection in pregnant women often leads to infant mortality or premature birth [5,57,58]. Delhi’s post-epidemic study found that HEV infection during pregnancy resulted in stillbirths, neonatal death, or miscarriage in 56% of cases [56].

The possibility of complications resulting from HEV infection during pregnancy depends on several factors, such as viral load, virus genotype, hormonal factors, and immune status [59,60]. Hepatitis E virus infection during pregnancy can be transmitted vertically from mother to child. It can have severe consequences for both mother and child, such as fulminant hepatic failure, to the mother and child’s death [5,61]. The risk is particularly pronounced in the third trimester of pregnancy, especially if the HEV-1 genotype causes the infection. In that case, maternal mortality is from 15% to 25% [62]. The mechanisms of liver damage in pregnant women caused by HEV infection are unknown [63,64]. However, based on research conducted, it is thought that the link is immunity and hormone level changes in pregnant women and viral factors, such as heterogeneity and variation in the HEV genome [11,65]. Pregnancy is an immune condition in which pregnant women are prone to developing viral infections [66].

Acute viral infection in pregnancy is associated with a wide range of harmful consequences for mother and fetus. The fetus can be affected by growth restriction, developmental abnormalities, premature birth, and stillbirth [67]. Mothers are predisposed to increased morbidity, primarily due to the development of acute liver failure (defined as the onset of jaundice, hepatic coagulopathy, and hepatic encephalopathy), which may eventually end in maternal death [68,69,70]. Pregnancy is likely to cause suppression of T cell-mediated immunity (including suppression of CD4 cells) and increased production of steroid hormones, leading to increased virus replication [71,72]. Upon entering the body, the virus can be present without visible antigens in a partially enveloped phase, which enables it to hide its existence from opsonizing antibodies. Likewise, infected cells reject many modulated ORF2 particles to complicate viral neutralization by antibodies even further [55]. Moreover, certain enzymes and cytokines such as TGF-β, interleukin 4, and interleukin 10 are secreted from placental and trophoblast cells [55]. This in turn suppresses cellular immunity at the circulation interface, between embryonic tissue and the uterine lining (maternal–fetal interface) and allows the transmission of HEV. Moreover, there are reports of HEV replicating within the placenta [71,73]. Therefore, rapid, effective response to invasive pathogens is vital to avoid massive maternal infection and consequent fetal complications [67].

### 4.1. Clinical Presentation of Hepatitis E in Pregnant Women Depending on the HEV Genotype

Different pathogenicity at the mother and fetus interface depending on HEV genotypes has been demonstrated by many studies [68]. Increased apoptosis and necrosis at the maternal–fetal interface are associated with changes in the placental barrier structure in HEV-1 and HEV-2 genotypes [68,74]. Consequently, higher morbidity and mortality in pregnant women infected with the HEV virus were predominantly observed in countries dominated by the HEV-1 genotype [55,75].

HEV infection in pregnant women often leads to infant mortality or premature birth, which makes it a critical, but hitherto mostly neglected, global public health problem [76].

The HEV-3 genotype has caused infections in developed countries, most notably France, Germany, and the United Kingdom. In those cases, no severe clinical features of HEV infection developed during pregnancy [55]. There is a possibility that maternal immune system factors may limit HEV-3 replication at the maternal–fetal interface, avoiding pregnancy complications [67]. The HEV-4 genotype is more dangerous than HEV-3 genotype infection and can lead to miscarriage and stillbirth [56]. In China, adverse pregnancy outcomes have been reported as a result of HEV-4 genotype infection and included premature rupture of the membrane, premature birth, and miscarriage [67].

### 4.2. Prevention Measures of HEV Infections in Pregnant Women

Prevention measures include enhanced hygiene measures, especially when consuming food and water in endemic regions [11]. A prophylactic recombinant vaccine against HEV infections has been approved in China [77], but implementation data is still lacking. Although anti-HEV antibodies and HEV RNA are present in the colostrum of mothers infected with HEV, breastfeeding appears to be safe for these infants [78]. However, there is a possibility of vertical transmission of HEV from infected mothers to their newborns. Accordingly, mothers should be advised to feed the newborn with formula in such situations [79,80].

## 5. Laboratory Diagnostics of Hepatitis E Virus

The first laboratory tests used to diagnose HEV infection cover electron microscopy, fluorescent antibody assay, and HEV antigen detection in hepatocytes. These methods relied on light microscopy and were time-consuming and technically demanding processes, thus were not amenable for HEV’s routine diagnosis. HEV genome cloning has enabled the development of molecular and serological assays to detect HEV infection [16]. There are two approaches to diagnose HEV infection: serological tests and nucleic acid tests. Serological tests are based on detecting either viral capsid antigens, or immunoglobulins (anti-HEV antibodies) produced by the immune system in response to HEV particles [50,81,82]. The sensitivity of these tests is typically high, but the specificity is rather low. Nucleic acid tests are based on detecting HEV RNA in the blood; these tests usually have high specificity and low sensitivity [17,83].

### 5.1. Serological Tests

The serological tests used to detect HEV infection belong to the group of enzyme immunoassay (EIA) tests. Indirect EIA assays detect IgM and IgG anti-HEV antibodies. IgM anti-HEV antibodies are the first ones to appear, approximately four weeks after the infection, and can be detected up to six months later. The presence of IgM anti-HEV antibodies implies that the HEV infection is acute or recent. IgG anti-HEV antibodies appear early after infection, almost simultaneously with IgM antibodies, and last for years. The presence of IgG anti-HEV antibodies indicates a recent or past infection [14,81,84,85]. Enzyme-linked immunosorbent assay (ELISA) is routinely used to detect IgM and IgG anti-HEV antibodies [12,16,86,87,88]. The problem with these assays is their variation in sensitivity and specificity, as shown in Table 3. Furthermore, the potential cross-reactivity of IgM with other viruses, such as cytomegalovirus (CMV) and Epstein–Barr virus (EBV), can be a problem [16,89,90]. The most commonly used commercial ELISA test for IgM detection is the Wantai test, which has the highest sensitivity and specificity among EIA tests [12,91].

Immunoblot assays have nitrocellulose strips and are a more prolonged process than ELISA. However, immunoblot assays are used as confirmatory tests for IgG anti-HEV antibodies [12,16,84].

Rapid immunochromatographic assays detect IgM anti-HEV antibodies. These are technically simpler and cheaper tests than EIA assays. Immunochromatographic assays are highly sensitive and specific and have a higher sensitivity than ELISA assays [12,17]. Such rapid tests are useful in pregnant women with suspected HEV infection due to the immediate need for treatment [81,92].

In addition to serological assays for detecting anti-HEV antibodies, there are also serological assays to detect viral antigens. Viral antigens are present in the early phase of acute HEV infection [12,16,17]; hence, this type of approach represents a direct method of detecting HEV infection based on the detection of capsid protein (ORF2) or phosphoprotein (ORF3) during acute infection. The ELISA assays are most commonly used to detect viral antigens in the blood or serum. These serological assays are simpler and cheaper than nucleic acid tests (NAT). The advantage of these assays is the detection of viral antigens before the onset of symptoms. However, the problem with these assays, as with other EIA assays, is their sensitivity and specificity. The commercial Wantai test for the detection of ORF2 antigen has high specificity and sound sensitivity [15,89].

### 5.2. Nucleic Acid Tests

HEV RNA could be detected in the blood and stool approximately three weeks after infection and shortly before the first symptoms appear. The nucleic acid test (NAT) is the gold standard for the diagnosis of HEV infection. The method is based on polymerase chain reaction (PCR) and its variants. The disadvantages of NAT methods are high costs, specialized devices, and staff expertise—all of which are limited in high burden and low income countries [23,93,94,95]. NAT methods have higher specificity and sensitivity compared to EIA, as shown in Table 3. The most commonly used are reverse transcription PCR (RT-PCR), multiplex PCR, real-time PCR (qPCR), nested PCR (nPCR), digital droplet PCR (ddPCR), and loop-mediated isothermal amplification assay (LAMP). HEV genome sequencing is less commonly used. Detection of RNA HEV is based on detecting the conserved ORF2/ORF3 region of the genome in four major HEV genotypes that infect humans. Real-time PCR works on the same principle as regular PCR, but is more specific and sensitive [13,50,84,96].

The LAMP test based on isothermal amplification is faster than PCR and does not require special equipment [15,97].

HEV genome sequencing begins with reverse transcription of HEV RNA, followed by nested PCR of a specific region and then Sanger sequencing. Typically, the PCR products of the ORF1 and ORF2 regions of the HEV are sequenced [12,15,98]. However, RT-PCR is more sensitive than nested PCR, and therefore sequencing is not used for diagnostic purposes [99], as shown in Table 3. Droplet digital PCR is a more sensitive method than qPCR for HEV RNA quantification because there is no need for a standard curve. Hence, it is more accurate in quantifying HEV RNA [96].

Many asymptomatic blood donors have HEV RNA detected by commercial HEV RNA tests [15]. A combination of serological assays and NAT is required to diagnose HEV infection correctly. In immunocompromised patients, serological assays of IgM anti-HEV antibody detection are not reliable. Therefore, the diagnosis must be based on the detection of HEV RNA in such patients [14,23,93].

**Figure 3:**
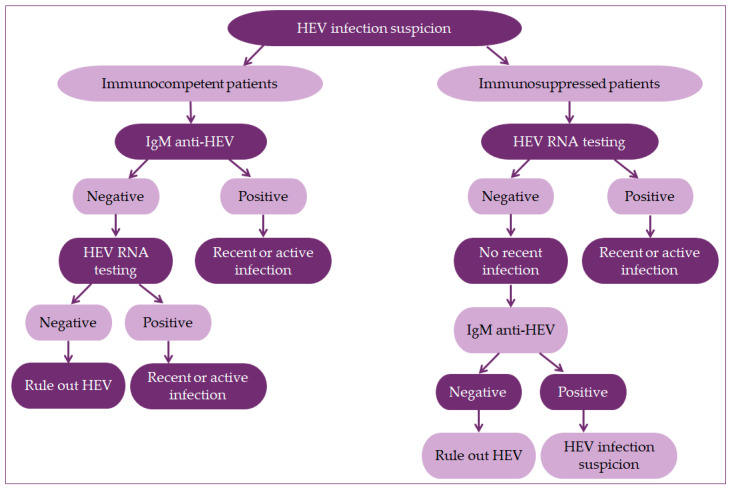
Algorithm for HEV infection diagnostic. Combining serological and nucleic acid testing is the best. The negative PCR test does not exclude the recent infection. Serology assays are sometimes negative in immunocompromised patients. Both negative test results rule out the HEV infection.

Due to diagnostic tests’ unreliability to detect HEV infection, the WHO has developed the first standard protocol for diagnosing four HEV genotypes that infect humans [13]. The recommendation is to test the presence of IgM anti-HEV antibodies initially. If the test is positive, the infection is either active or recent, and a molecular test to detect HEV RNA can be performed [14,50,85]. In immunocompromised patients, a positive test result confirms HEV infection. Only if both test results (i.e., IgM anti-HEV antibodies and HEV RNA) are negative, HEV infection is excluded, as shown in Figure 3. Such combination of serological and molecular tests increases the specificity and sensitivity, and thus opens the door for accurate and timely diagnosis of HEV infection [81,92,100]. This is especially important in asymptomatic blood donors and organ donors, but also a substantial population of pregnant women who could have significant health consequences due to the untimely diagnosis of HEV infection [14,81,101,102].

Considering the latter, Zhao et al. pursued a cost-effectiveness analysis on pregnant women in epidemic regions of the world and showed that the combined approach of screening and vaccination in women of childbearing age had higher quality-adjusted life-year (QALY) and lower costs in comparison to the universal vaccination only (as in this case, only women negative for HEV antibodies would be vaccinated) [103]. Moreover, the ease of testing for capsid antigen (together with much lower cost in comparison to RNA detection) makes it a viable option not only for blood screening, but also in establishing an early HEV diagnosis in pregnancy [17]. Akin to the Markov cohort model that demonstrated the cost-effectiveness of routine HEV screening in solid organ transplant patients [104], similar studies are needed for the population of pregnant women, but also other vulnerable groups.

## 6. Conclusions

Hepatitis E infections are increasingly present both in and outside endemic regions. They are most often zoonoses, although cases of transfusion transmission have been reported, and they occur in both low and highly developed countries. As pregnant women are the most vulnerable and at-risk group, it is necessary to introduce preventive measures that should include screening for HEV RNA—especially if we take into account that there is still no adequate therapy for pregnant women. Moreover, guidelines for the control of HEV infections in pregnancy are still lacking, which means there is a need to fill the gaps in the literature by additional research endeavors and consensus approaches. It is also essential to investigate HEV genotypes’ prevalence according to their virulence and morbidity in pregnancy. In addition, while the hepatitis E vaccine is being tested for safety in this population and safer treatment approaches are being considered, it is pivotal to adhere to all the necessary prevention measures in the meantime, but also to test pregnant women (especially in endemic areas) and assess the risk for pregnancy on a case-to-case basis.

## Figures and Tables

**Table 1:** Classification of hepatitis E virus (HEV).

Genus	*Orthohepevirus*
Family	*Hepeviridae*
Species	A, B, C, D
Genotypes	1,2,3,4,5,6,7,8
Virion	32–34 nm, icosahedral
Envelope	quasi-enveloped
Genome	single-stranded, positive-sense, linear RNA
Genome size	7.2 kb
Sensitivity	Inactivation at temperatures above 70 °C

**Table 2:** Distribution of HEV genotypes.

HEV Genotype	Host	Endemic Regions
1	Human	Asia, North Africa, Mexico, Middle East
2	Human	Asia, North Africa, Mexico, Middle East
3	Human, rabbit, pig, mongoose	Worldwide
4	Human, domestic pig, and wild boar	South and East Asia, the United States, Europe, China, and Japan
5	Wild boar	Japan
6	Wild boar	Japan
7	Camel	Dubai
8	Camel	Dubai, western China

**Table 3:** The advantages and disadvantages of serological assays and the nucleic acid test for HEV diagnostic purposes.

	Method	Type of Molecule Detected	Advantages	Disadvantages
**Serological tests**	Enzyme immunoassay (EIA)	IgM and/or IgG anti-HEV antibodies	Quick, easy to perform, less expensive than a molecular test, high sensitivity, low specificity, epidemiological surveys	Discrepancies in the results, vary in sensitivity, specificity, false-positive reaction through cross-reactive antibodies
		Viral capsid antigen and phosphoprotein	Technically less demanding than PCR	Antigens disappear quickly with seroconversion, low sensitivity, the poor performance of polypeptides, limited application, less sensitivity than real-time PCR
	Rapid immunochromatographic assays	IgM anti-HEV antibodies	Sensitivity comparable with PCR, higher than ELISA, fast, simple, point of care testing	Low accuracy in detecting other genotypes than genotype 3
**Nucleic acid tests**	Reversion transcription and PCR (RT-PCR)	ORF2 region	High sensitivity and specificity, identification of HEV genotypes	Low levels of HEV RNA before symptoms, a brief period of viremia
	Nested PCR	ORF2 region	High specificity and sensitivity for genotype 1	The complicated procedure, strict requirements, contamination
	Real-time PCR (qPCR)	ORF2 and ORF3 region	High specificity, high sensitivity, more sensitive than nPCR, simple operation, quantification	Trained staff, test facilities, high cost in developing countries, polymorphisms in primer site results with false-negative results
	Loop-mediated isothermal amplification assay (LAMP)	ORF3 region	Fast, no special equipment, high sensitivity, and efficiency	Limited experience, works for genotypes 1 and 4, no data for other genotypes
	RNA sequencing	ORF1 and ORF2 region	Accurate genotyping	Variability, lack of standardization, high cost, polymorphisms in primer site results in false-negative results

## Data Availability

No new data were created or analyzed in this study. Data sharing is not applicable to this article.

## References

[1] LeDesma R., Nimgaonkar I., Ploss A. (2019). Hepatitis E Virus Replication. Viruses.

[2] Emerson S.U., Purcell R.H. (2003). Hepatitis E virus. Rev. Med. Virol..

[3] Lhomme S., Marion O., Abravanel F., Izopet J., Kamar N. (2020). Clinical Manifestations, Pathogenesis and Treatment of Hepatitis E Virus Infections. J. Clin. Med..

[4] Sridhar S., Teng J.L.L., Chiu T.H., Lau S.K.P., Woo P.C.Y. (2017). Hepatitis E virus genotypes and evolution: Emergence of camel hepatitis E variants. Int. J. Mol. Sci..

[5] Chaudhry S.A., Verma N., Koren G. (2015). Hepatitis E infection during pregnancy. Can. Fam. Physician.

[6] Fousekis F.S., Mitselos I.V., Christodoulou D.K. (2020). Extrahepatic manifestations of hepatitis E virus: An overview. Clin. Mol. Hepatol..

[7] Nair V.P., Anang S., Subramani C., Madhvi A., Bakshi K., Srivastava A., Shalimar, Nayak B., Kumar C.T.R., Surjit M. (2016). Endoplasmic Reticulum Stress Induced Synthesis of a Novel Viral Factor Mediates Efficient Replication of Genotype-1 Hepatitis E Virus. PLoS Pathog..

[8] Meng X.J. (2010). Recent advances in Hepatitis E Virus. J. Viral Hepat..

[9] Goel A., Aggarwal R. (2020). Hepatitis E: Epidemiology, Clinical Course, Prevention, and Treatment. Gastroenterol. Clin. N. Am..

[10] Yin X., Feng Z. (2019). Hepatitis E Virus Entry. Viruses.

[11] Wu C., Wu X., Xia J. (2020). Hepatitis E virus infection during pregnancy. Virol. J..

[12] Al-Sadeq D.W., Majdalawieh A.F., Mesleh A.G., Abdalla O.M., Nasrallah G.K. (2018). Laboratory challenges in the diagnosis of hepatitis E virus. J. Med. Microbiol..

[13] Pallerla S.R., Harms D., Johne R., Todt D., Steinmann E., Schemmerer M., Wenzel J.J., Hofmann J., Shih J.W.K., Wedemeyer H. (2020). Hepatitis E Virus Infection: Circulation, Molecular Epidemiology, and Impact on Global Health. Pathogens.

[14] Aslan A.T., Balaban H.Y. (2020). Hepatitis E virus: Epidemiology, diagnosis, clinical manifestations, and treatment. World J. Gastroenterol..

[15] Lhomme S., Legrand-Abravanel F., Kamar N., Izopet J. (2019). Screening, diagnosis and risks associated with Hepatitis E virus infection. Expert Rev. Anti-Infect. Ther..

[16] Khudyakov Y., Kamili S. (2011). Serological diagnostics of hepatitis E virus infection. Virus Res..

[17] Kar P., Karna R. (2020). A Review of the Diagnosis and Management of Hepatitis E. Curr. Treat. Options Infect. Dis..

[18] Nan Y., Zhang Y.J. (2016). Molecular biology and infection of hepatitis E virus. Front. Microbiol..

[19] Oechslin N., Moradpour D., Gouttenoire J. (2020). On the Host Side of the Hepatitis E Virus Life Cycle. Cells.

[20] Kenney S.P., Meng X.J. (2019). Hepatitis E virus genome structure and replication strategy. Cold Spring Harb. Perspect. Med..

[21] Himmelsbach K., Bender D., Hildt E. (2018). Life cycle and morphogenesis of the hepatitis E virus. Emerg. Microbes Infect..

[22] Debing Y., Moradpour D., Neyts J., Gouttenoire J. (2016). Update on hepatitis E virology: Implications for clinical practice. J. Hepatol..

[23] Ahmad T., Nasir S., Musa T.H., AlRyalat S.A.S., Khan M., Hui J. (2020). Epidemiology, diagnosis, vaccines, and bibliometric analysis of the 100 top-cited studies on Hepatitis E virus. Hum. Vaccines Immunother..

[24] Panda S.K., Varma S.P.K. (2013). Hepatitis E: Molecular Virology and Pathogenesis. J. Clin. Exp. Hepatol..

[25] Ju X., Ding Q. (2019). Hepatitis E virus assembly and release. Viruses.

[26] Thakur V., Ratho R.K., Kumar S., Saxena S.K., Bora I., Thakur P. (2020). Viral Hepatitis E and Chronicity: A Growing Public Health Concern. Front. Microbiol..

[27] Sayed I.M., Vercouter A.S., Abdelwahab S.F., Vercauteren K., Meuleman P. (2015). Is hepatitis E virus an emerging problem in industrialized countries?. Hepatology.

[28] World Health Organization Hepatitis E. https://www.who.int/news-room/fact-sheets/detail/hepatitis-e.

[29] Teshale E., Brunette G., Nemhauser J. (2019). Travel-Related Infectious Diseases. Yellow Book.

[30] Horvatits T., Schulze zur Wiesch J., Lütgehetmann M., Lohse A.W., Pischke S. (2019). The Clinical Perspective on Hepatitis E. Viruses.

[31] Denner J. (2019). Hepatitis e virus (HEV)—The future. Viruses.

[32] Clemente-Casares P., Ramos-Romero C., Ramirez-Gonzalez E., Mas A. (2016). Hepatitis E virus in industrialized countries: The silent threat. Biomed. Res. Int..

[33] Primadharsini P.P., Nagashima S., Okamoto H. (2019). Genetic variability and evolution of hepatitis E virus. Viruses.

[34] Gupta E., Agarwala P. (2018). Hepatitis E virus infection: An old virus with a new story!. Indian J. Med. Microbiol..

[35] Hakim M.S., Wang W., Bramer W.M., Geng J., Huang F., de Man R.A., Peppelenbosch M.P., Pan Q. (2017). The global burden of hepatitis E outbreaks: A systematic review. Liver Int..

[36] Teshale E.H., Hu D.J., Holmberg S.D. (2010). The two faces of hepatitis E virus. Clin. Infect. Dis..

[37] Narayanan S., Abutaleb A., Sherman K.E., Kottilil S. (2019). Clinical features and determinants of chronicity in hepatitis E virus infection. J. Viral Hepat..

[38] Sooryanarain H., Meng X.J. (2019). Hepatitis E virus: Reasons for emergence in humans. Curr. Opin. Virol..

[39] Blasco-Perrin H., Abravanel F., Blasco-Baque V., Péron J.M. (2016). Hepatitis E, the neglected one. Liver Int..

[40] Carratalà A., Joost S. (2019). Population density and water balance influence the global occurrence of hepatitis E epidemics. Sci. Rep..

[41] Wallace S.J., Crossan C., Hussaini S.H., Dalton H.R. (2019). Hepatitis E: A largely underestimated, emerging threat. Br. J. Hosp. Med..

[42] Wedemeyer H., Pischke S., Manns M.P. (2012). Pathogenesis and treatment of hepatitis E virus infection. Gastroenterology.

[43] Sclair S.N., Schiff E.R. (2013). An update on the hepatitis E virus. Curr. Gastroenterol. Rep..

[44] Takova K., Koynarski T., Minkov I., Ivanova Z., Toneva V., Zahmanova G. (2020). Increasing Hepatitis E Virus Seroprevalence in Domestic Pigs and Wild Boar in Bulgaria. Animals.

[45] Khuroo M.S., Khuroo M.S., Khuroo N.S. (2016). Hepatitis E: Discovery, global impact, control and cure. World J. Gastroenterol..

[46] Wang Y., Wang S., Wu J., Jiang Y., Zhang H., Li S., Liu H., Yang C., Tang H., Guo N. (2018). Hepatitis E virus infection in acute non-traumatic neuropathy: A large prospective case-control study in China. EBioMedicine.

[47] Cong W., Sui J.C., Zhang X.Y., Qian A.D., Chen J., Zhu X.Q. (2015). Seroprevalence of hepatitis E virus among pregnant women and control subjects in China. J. Med. Virol..

[48] Ricci A., Allende A., Bolton D., Chemaly M., Davies R., Fernandez Escamez P.S., Herman L., Koutsoumanis K., Lindqvist R., Nørrung B. (2017). Public health risks associated with hepatitis E virus (HEV) as a food-borne pathogen. EFSA J..

[49] Ijaz S., Vyse A.J., Morgan D., Pebody R.G., Tedder R.S., Brown D. (2009). Indigenous hepatitis E virus infection in England: More common than it seems. J. Clin. Virol..

[50] Webb G.W., Dalton H.R. (2019). Hepatitis E: An underestimated emerging threat. Ther. Adv. Infect. Dis..

[51] Đaković Rode O., Jemeršić L., Vince A. (2016). Hepatitis E in Croatia-Guidelines for Diagnosis and Treatment. Liječnički Vjesn..

[52] Jemeršić L., Keros T., Maltar L., Barbić L., Čavlek T.V., Jeličić P., Rode O.Đ., Prpić J. (2017). Differences in hepatitis E virus (HEV) presence in naturally infected seropositive domestic pigs and wild boars—An indication of wild boars having an important role in HEV epidemiology. Vet. Arh..

[53] Mrzljak A., Dinjar-Kujundzic P., Jemersic L., Prpic J., Barbic L., Savic V., Stevanovic V., Vilibic-Cavlek T. (2019). Epidemiology of hepatitis e in South-East Europe in the “one Health” concept. World J. Gastroenterol..

[54] Jemeršić L., Prpić J., Brnić D., Keros T., Pandak N., Rode O.Đ. (2019). Genetic diversity of hepatitis E virus (HEV) strains derived from humans, swine and wild boars in Croatia from 2010 to 2017. BMC Infect. Dis..

[55] Nelson K.E., Labrique A.B., Kmush B.L. (2019). Epidemiology of genotype 1 and 2 hepatitis E virus infections. Cold Spring Harb. Perspect. Med..

[56] Abebe M., Ali I., Ayele S., Overbo J., Aseffa A., Mihret A. (2017). Seroprevalence and risk factors of Hepatitis E Virus infection among pregnant women in Addis Ababa, Ethiopia. PLoS ONE.

[57] Kmush B.L., Nelson K.E., Labrique A.B. (2015). Risk factors for hepatitis E virus infection and disease. Expert Rev. Anti-Infect. Ther..

[58] Bergløv A., Hallager S., Weis N. (2019). Hepatitis E during pregnancy: Maternal and foetal case-fatality rates and adverse outcomes—A systematic review. J. Viral Hepat..

[59] El-Mokhtar M.A., Othman E.R., Khashbah M.Y., Ismael A., Ghaliony M.A., Seddik M.I., Sayed I.M. (2020). Evidence of the Extrahepatic Replication of Hepatitis E Virus in Human Endometrial Stromal Cells. Pathogens.

[60] Wedemeyer H., Rybczynska J., Pischke S., Krawczynski K. (2013). Immunopathogenesis of hepatitis E virus infection. Semin. Liver Dis..

[61] Xin S., Xiao L. (2016). Clinical manifestations of hepatitis E. Adv. Exp. Med. Biol..

[62] Yu W., Hao X., Li Y., Yang C., Li Y., He Z., Huang F. (2020). Vertical transmission of hepatitis E virus in pregnant rhesus macaques. Sci. Rep..

[63] Racicot K., Mor G. (2017). Risks associated with viral infections during pregnancy. J. Clin. Invest..

[64] Sookoian S. (2006). Liver disease during pregnancy: Acute viral hepatitis. Ann. Hepatol..

[65] Abravanel F., Lhomme S., Dubois M., Peron J.M., Alric L., Kamar N., Izopet J. (2013). Hepatitis E virus. Med. Mal. Infect..

[66] Cornish E.F., Filipovic I., Åsenius F., Williams D.J., McDonnell T. (2020). Innate Immune Responses to Acute Viral Infection During Pregnancy. Front. Immunol..

[67] Gouilly J., Chen Q., Siewiera J., Cartron G., Levy C., Dubois M., Al-Daccak R., Izopet J., Jabrane-Ferrat N., El Costa H. (2018). Genotype specific pathogenicity of hepatitis E virus at the human maternal-fetal interface. Nat. Commun..

[68] Seth A., Sherman K.E. (2020). Hepatitis E: What We Think We Know. Clin. Liver Dis..

[69] Aggarwal R.A. (2013). Hepatitis E: Clinical presentation in disease-endemic areas and diagnosis. Semin. Liver Dis..

[70] Shalimar, Acharya S.K. (2013). Hepatitis E and Acute Liver Failure in Pregnancy. J. Clin. Exp. Hepatol..

[71] Julin C.H., Hjortaas K., Dembinski J.L., Sandbu S., Øverbø J., Stene-Johansen K., Dudman S. (2019). Hepatitis E in Pregnant Women and the Potential Use of HEV Vaccine to Prevent Maternal Infection and Mortality. Curr. Trop. Med. Rep..

[72] Navaneethan U., Al Mohajer M., Shata M.T. (2008). Hepatitis E and pregnancy: Understanding the pathogenesis. Liver Int..

[73] Rac M.W.F., Sheffield J.S. (2014). Prevention and management of viral hepatitis in pregnancy. Obstet. Gynecol. Clin. N. Am..

[74] Aggarwal R., Naik S. (2009). Epidemiology of hepatitis E: Current status. J. Gastroenterol. Hepatol..

[75] Terrault N.A., Levy M.T., Cheung K.W., Jourdain G. (2020). Viral hepatitis and pregnancy. Nat. Rev. Gastroenterol. Hepatol..

[76] Yang C., Hao X., Li Y., Long F., He Q., Huang F., Yu W. (2019). Successful establishment of hepatitis E virus infection in pregnant BALB/c mice. Viruses.

[77] Li Y., Huang X., Zhang Z., Li S., Zhang J., Xia N., Zhao Q. (2020). Prophylactic Hepatitis E Vaccines: Antigenic Analysis and Serological Evaluation. Viruses.

[78] Chibber R.M., Usmani M.A., Al-Sibai M.H. (2004). Should HEV infected mothers breast feed?. Arch. Gynecol. Obstet..

[79] Aggarwal R., Jameel S. (2011). Hepatitis E. Hepatology.

[80] Krain L.J., Atwell J.E., Nelson K.E., Labrique A.B. (2014). Fetal and neonatal health consequences of vertically transmitted hepatitis E virus infection. Am. J. Trop. Med. Hyg..

[81] Kar P., Sengupta A. (2019). A guide to the management of hepatitis E infection during pregnancy. Expert Rev. Gastroenterol. Hepatol..

[82] Webb G.W., Kelly S., Dalton H.R. (2020). Hepatitis A and Hepatitis E: Clinical and Epidemiological Features, Diagnosis, Treatment, and Prevention. Clin. Microbiol. Newsl..

[83] Taherkhani R., Farshadpour F. (2016). Epidemiology of hepatitis E in pregnant women and children in Iran: A general overview. J. Clin. Transl. Hepatol..

[84] Dalton H.R., Kamar N., Baylis S.A., Moradpour D., Wedemeyer H., Negro F., European Association for the Study of the Liver (2018). EASL Clinical Practice Guidelines on hepatitis E virus infection. J. Hepatol..

[85] Zhao C., Wang Y., Wang Y. (2016). Laboratory diagnosis of HEV infection. Hepatitis E Virus. Advances in Experimental Medicine and Biology.

[86] Candido A., Taffon S., Chionne P., Pisani G., Madonna E., Dettori S., Hamza A., Valdarchi C., Bruni R., Ciccaglione A.R. (2012). Diagnosis of HEV infection by serological and real-time PCR assays: A study on acute non-A-C hepatitis collected from 2004 to 2010 in Italy. BMC Res. Notes.

[87] Marrone G., Biolato M., Mercurio G., Capobianchi M.R., Garbuglia A.R., Liguori A., Vassallo G., Gasbarrini A., Miele L., Grieco A. (2019). Acute HEV hepatitis: Clinical and laboratory diagnosis. Eur. Rev. Med. Pharmacol. Sci..

[88] Myint K.S.A., Endy T.P., Gibbons R.V., Laras K., Mammen M.P., Sedyaningsih E.R., Seriwatana J., Glass J.S., Narupiti S., Corwin A.L. (2006). Evaluation of diagnostic assays for hepatitis E virus in outbreak settings. J. Clin. Microbiol..

[89] Aggarwal R. (2013). Diagnosis of hepatitis E. Nat. Rev. Gastroenterol. Hepatol..

[90] Doting M.H.E., Weel J., Niesters H.G.M., Riezebos-Brilman A., Brandenburg A. (2017). The added value of hepatitis E diagnostics in determining causes of hepatitis in routine diagnostic settings in the Netherlands. Clin. Microbiol. Infect..

[91] Sampedro A., Casanovas I., Ceballos J., Rodriguez-Granger J., Cobo F., Navarro J.M. (2020). Comparative evaluation of two immunoassays for serological diagnosis of hepatitis E. J. Med. Virol..

[92] Pérez-Gracia M.T., Suay-García B., Mateos-Lindemann M.L. (2017). Hepatitis E and pregnancy: Current state. Rev. Med. Virol..

[93] Larrue H., Abravanel F., Péron J. (2020). Hepatitis E, what’s the real issue?. Liver Int..

[94] Prasidthrathsint K., Stapleton J.T. (2019). Laboratory Diagnosis and Monitoring of Viral Hepatitis. Gastroenterol. Clin. N. Am..

[95] Mirazo S., Ramos N., Mainardi V., Arbiza J., Gerona S. (2014). Transmission, diagnosis, and management of hepatitis E: An update. Hepatic Med. Evid. Res..

[96] Nicot F., Cazabat M., Lhomme S., Marion O., Sauné K., Chiabrando J., Dubois M., Kamar N., Abravanel F., Izopet J. (2016). Quantification of HEV RNA by droplet digital PCR. Viruses.

[97] Lan X., Yang B., Bao Y.L., Xiang P.Y., Xue R.L., Ji X.L. (2009). Reverse transcription-loop-mediated isothermal amplification assay for rapid detection of hepatitis E virus. J. Clin. Microbiol..

[98] European Centre for Disease Prevention and Control (2019). Options for National Testing and Surveillance for Hepatitis E virus in the EU/EEA—Operational Guidance.

[99] Rivero Juárez A. (2018). Consensus Document of the Diagnosis, Management, and Prevention of Infection with the Hepatitis E virus: Study Group for Viral Hepatitis (GEHEP) of the Spanish Society of Infectious Diseases and Clinical Microbiology (SEIMC). http://www.seimc.org.

[100] Anastasiou O.E., Thodou V., Berger A., Wedemeyer H., Ciesek S. (2020). Comprehensive evaluation of hepatitis E serology and molecular testing in a large cohort. Pathogens.

[101] Desai A.N. (2020). Hepatitis E. JAMA.

[102] Bi H., Yang R., Wu C., Xia J. (2020). Hepatitis E Virus and Blood Transfusion Safety. Epidemiol. Infect..

[103] Zhao Y., Zhang X., Zhu F., Jin H., Wang B. (2016). A preliminary cost-effectiveness analysis of hepatitis E vaccination among pregnant women in epidemic regions. Hum. Vaccin. Immunother..

[104] Ankcorn M.J., Tedder S.R., Cairns J., Sandmann F.G. (2020). Cost-Effectiveness Analysis of Screening for Persistent Hepatitis E Virus Infection in Solid Organ Transplant Patients in the United Kingdom: A Model-Based Economic Evaluation. Value Health.

